# Damage-Free Vertical Microfabrication of α‑Quartz via HF Gas-Phase Catalyst Etching

**DOI:** 10.1021/acsami.5c10265

**Published:** 2025-08-16

**Authors:** Ko-hei Sano, Yoshitaka Ono, Keishi Tsukiyama, Sho Nagai, Atsushi Saito, Yasuo Hayashi, Takahiko Yanagitani

**Affiliations:** † Graduate School of Advanced Science and Engineering, 13148 Waseda University, Tokyo 169-8555, Japan; ‡ Innovative Technology Laboratories, 53325 AGC Incorporated, Kanagawa 230-0045, Japan; § Kagami Memorial Research Institute for Material Science and Technology, 13148 Waseda University, Tokyo 169-0051, Japan

**Keywords:** Gas-Phase Catalyst Etching, Deep Microfabrication, Quartz Crystal, HF Gas, Damage Free

## Abstract

Vertical microfabrication of α-quartz is typically performed via reactive-ion etching; however, ion irradiation from a plasma source etches the hard mask and disrupts the crystal structure of the etched surface. Thus, the microstructures fabricated via the conventional process have limitations in terms of the taper angle, depth, and crystallinity, which limits technological innovation in quartz devices. In this study, we introduce a plasma-free gas-phase catalyst etching for α-quartz using HF gas and a patterned photoresist as a catalyst. This method realizes vertical etching without depth limitations, regardless of the crystal orientations because the etching reaction in the catalyst contact area on the quartz is dominant over the crystal anisotropy. Therefore, high-aspect-ratio deep microstructures were fabricated on the α-quartz without forming damage layers, e.g., amorphization or chemical-state changes. Our findings may lead to inventions in material and device design using microfabricated α-quartz.

## Graphical abstract

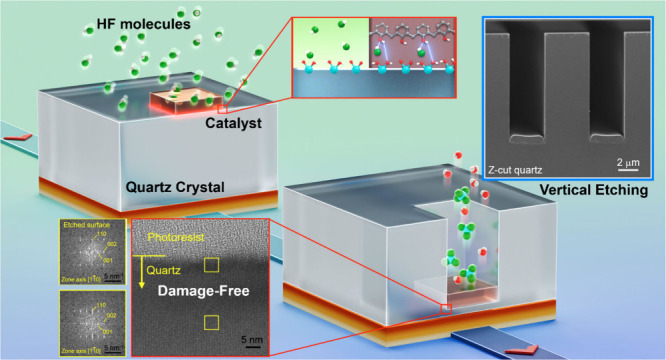

## Introduction

Quartz is a transparent hexagonal prismatic crystal composed of Si and O atoms and is a naturally occurring, mineable material. It has been known as a gemstone since ancient times; however, its piezoelectric properties were discovered by the Curie brothers in 1880,^1^ paving the way for the industrial use of α-quartz. Piezoelectric quartz resonators have since become essential in everyday life because they provide accurate clock signals for the stable operation of advanced systems such as cellphones, laptops, medical equipment, and automobiles.^2–5^ The performance of quartz resonators depends on the cutout angle of the crystal block relative to the crystal axis and the thickness of the quartz substrate. Therefore, 35.15° Y-rotated (AT-cut) thin plates with a zero temperature coefficient of frequency near room temperature (∼25 °C) are widely used in quartz resonators and quartz crystal microbalances.^6–16^ Single-crystal α-quartz with other cutout angles are also used in various applications. For example, X- and Y-cut quartz is used in optical waveplates,^17–19^ and 42.75° Y-rotated (ST-cut) quartz is used in surface acoustic wave resonators.^20,21^ In recent years, the use of microfabrication processes has been proposed to improve the performance of conventional flat-type quartz devices.^22–30^

Etching processes can be used to fabricate high-resolution microstructures on a substrate. The conventional method of wet chemical etching with HF or HF + NH_4_F solutions is inexpensive and suitable for mass production.^22,23,31–35^ However, the wet etching of α-quartz is an anisotropic process that depends on the crystal orientations. Therefore, the etched microstructures are limited by the crystal anisotropy of α-quartz. Reactive-ion etching (RIE) is a dry process typically used for vertical microfabrication.^24–28,36–39^ The etching reaction proceeds via ion irradiation to the etched surfaces that react with active species generated under the plasma conditions. Hence, vertical microstructures independent of the crystallographic orientations can be fabricated on the quartz. However, the ions also etch the hard masks on the substrate during the RIE process. Thus, the etched structure parameters, such as the taper angles and etching depth, are limited by the selectivity ratio of α-quartz and mask materials. Furthermore, accelerated ions and heat from the plasma source disrupt the crystal structure of the etched surface. Therefore, the realization of vertical deep etching without forming plasma-damage layers on α-quartz is challenging in microfabrication processes.

HF gas-phase surface treatment is a plasma-free dry etching method for SiO_2._ ^40–46^ The gas-phase etching reaction can be expressed as

$${\mathrm{SiO}}_{2}+4\mathrm{HF}\to {\mathrm{SiF}}_{4}+2H_{2}O$$

This gas-phase process is suitable for mass production because multiple substrates can be treated simultaneously. Furthermore, the etched surface is not damaged because the HF molecules are chemically etched without a plasma environment. However, the microstructures on α-quartz fabricated via HF gas-phase etching are anisotropic, depending on the crystal orientations.

Recently, a novel gas-phase vertical etching method for amorphous silica using HF gas and a patterned photoresist as catalysts was developed.^47^ The hydrophilic functional groups in the photoresist catalyze nucleophilic attacks on SiO_2_ by HF molecules under high-temperature conditions. Additionally, gas-phase HF molecules can pass through the photoresist to reach the SiO_2_ contact interface. This method achieves catalyst etching of SiO_2_, which could not be realized using a metal-assisted chemical etching process with a noble-metal catalyst.^48–54^ Therefore, vertical microstructures with smooth sidewalls can be formed on amorphous silica without the use of hard masks or ion irradiations. Furthermore, submicrometer-scale vertical microstructures with high aspect ratios can be fabricated because photoresists with fine high-resolution patterns are easily obtained using general lithography systems. As the gas-phase chemical etching reaction mainly proceeds in the photoresist-covered area on the substrate, we hypothesized that HF gas-phase catalyst etching could realize deep vertical microfabrication on α-quartz without forming damage layers.

This study verified that HF gas-phase catalyst etching can be used to fabricate vertical microstructures on α-quartz. The processing temperature was set below the α-β quartz phase transition temperature to prevent heat damage to the quartz during the process. As the HF gas-phase etching reaction occurred only in the catalyst-covered area on the α-quartz, vertical microfabrication independent of the crystallographic orientation was demonstrated. Additionally, in contrast to RIE, the HF gas-phase catalyst etching method was found to allow deep etching without a depth limitation. Furthermore, the etched surface fabricated via the catalyst etching did not show the amorphization and chemical-state changes that are typically caused by RIE.

## Results and Discussion

### HF Gas-Phase Catalyst Etching of α-Quartz

During HF gas-phase catalyst etching, the etching reaction mainly proceeds on the part of the substrate covered by the photoresist, regardless of the orientation of α-quartz. Therefore, vertical microfabrication independent of the orientation of the quartz can be achieved without using a plasma source and a hard mask, as shown in Figure 1a. To verify this aspect, HF gas-phase catalyst etching was performed on α-quartz with various crystal orientations. After HF gas-phase catalyst etching at 350 °C for 1000 s, vertical trench structures were observed on the Z-, X-, and AT-cut quartz substrates (Figure 1b–e). The photoresist used as a catalyst was detected at the bottom of the trenches after the catalyst etching (Figure 1f). Additionally, anisotropic microstructures dependent on the crystal orientations of the quartz were not observed on the bare quartz surfaces and at the bottom of the trenches after the catalyst etching, as shown in Figure 1g. Furthermore, a smooth surface was observed at the bottom of the trenches after removal of the catalyst. The surface roughness of the etched bottom areas on the quartz was evaluated by using atomic force microscopy (AFM). As shown in Figure 1h, the arithmetic mean roughness (Ra) values obtained from the AFM images of the etched bottoms were 2.7 and 3.6 nm for Z- and X-cut quartz, respectively. The surface roughness is thought to be transferred from the shape of the photoresist to the etched surface. Nanometer-scale fine particles were observed within the photoresist used as a catalyst, as shown in Figure 1i. The catalytic activity of the particles are considered to cause the formation of nanometer-scale roughness on the etched surfaces. The etched sidewalls also contact the photoresist during the process. Therefore, the Ra values of the etched sidewalls are expected to be of the same order as that of the bottom surfaces. These characteristics are fundamentally different from the microstructures of α-quartz fabricated via wet chemical etching, which exhibit a crystal orientation dependence. Therefore, the etching reaction in the catalyst contact area on the α-quartz was dominant over the crystal anisotropy, forming a vertical microstructure on the quartz.

**Figure 1:**
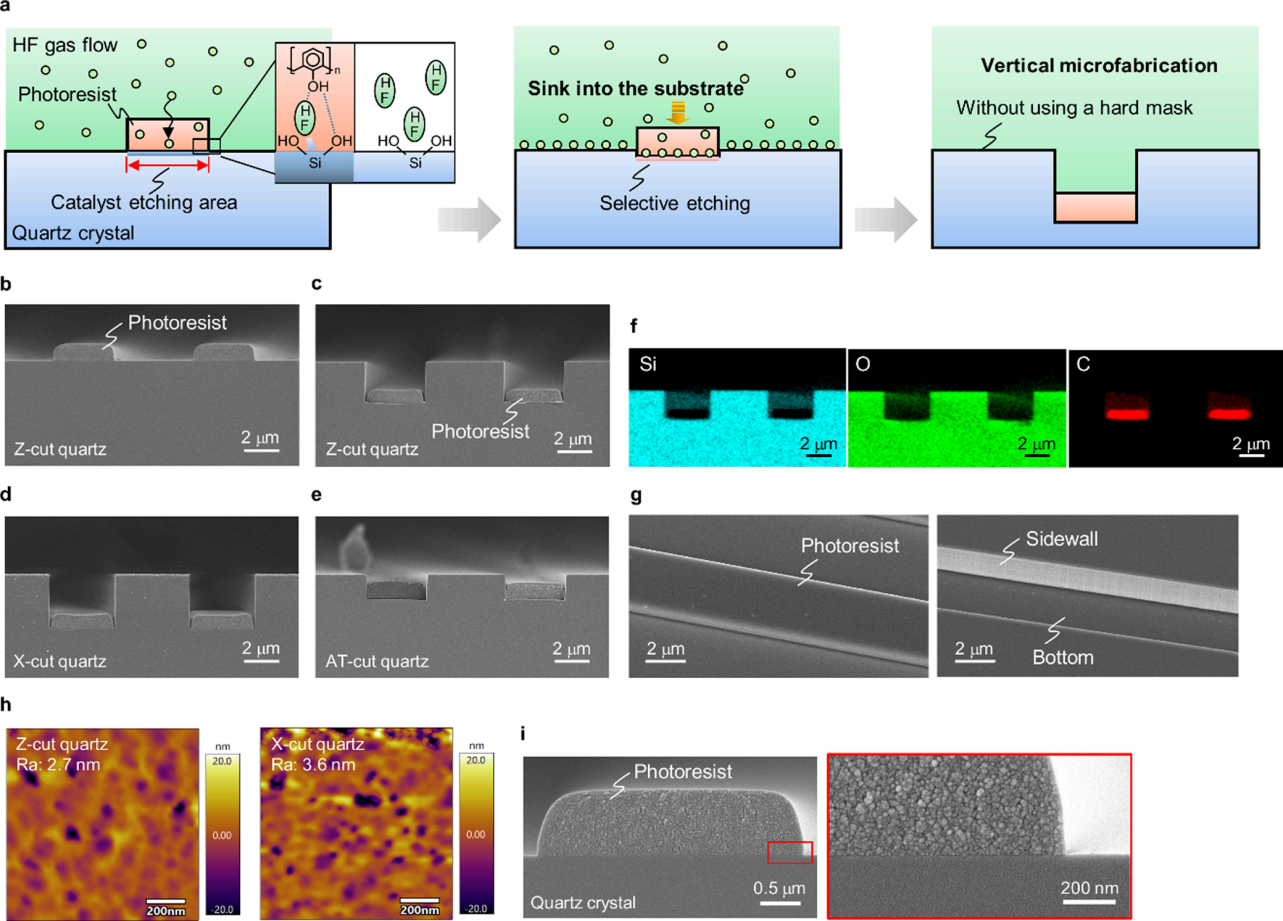
Microfabrication of α-quartz via HF gas-phase catalyst etching. (a) Schematic of vertical microfabrication via HF gas-phase catalyst etching. (b) Cross-sectional scanning electron microscopy (SEM) images of the photoresist pattern on Z-cut quartz before HF gas-phase catalyst etching. (c) Cross-sectional SEM images of the photoresist pattern on Z-cut quartz after HF gas-phase catalyst etching. (d) Cross-sectional SEM images of the photoresist pattern on X-cut quartz after HF gas-phase catalyst etching. (e) Cross-sectional SEM images of the photoresist pattern on AT-cut quartz after HF gas-phase catalyst etching. (f) Cross-sectional energy-dispersive X-ray spectroscopy (EDS) mapping images of Si, O, and C in the photoresist pattern on Z-cut quartz after HF gas-phase catalyst etching. (g) 30°-tilted SEM images of the photoresist pattern on Z-cut quartz before (left) and after (right) HF gas-phase catalyst etching. The photoresist was removed from the trenches after the catalyst etching. (h) Atomic force microscopy (AFM) images of the etched bottom surface on Z-cut quartz (left) and X-cut quartz (right) after removal of the photoresist. (i) Cross-sectional SEM image (left) and magnified SEM image (right) showing the photoresist pattern on the quartz before HF gas-phase catalyst etching.

The catalyst etching reaction with the α-quartz occurred below the α–β quartz phase transition temperature under atmospheric pressure conditions,^55,56^ as shown in Figure 2a. The etching rate was increased from 100 to 300 °C, and the maximum etching rates of the quartz were approximately the same among different crystallographic planes. However, the etching reaction was suppressed when the processing temperature was >300 °C. The novolac-type photoresist used as the catalyst contains hydroxyl groups that serve as active sites for the catalytic reaction.^47^ The number of hydroxyl groups in the photoresist is reduced upon increasing the processing temperature owing to thermal decomposition, as shown in Figure 2b. Consequently, the catalytic chemical reaction pathway was suppressed under high-temperature conditions. The maximum etching rates of α-quartz were significantly lower than those of silica glass. This difference is attributed to the chemical durability of α-quartz and silica glass. Quartz crystals and silica glass have tetrahedral structures composed of SiO_4_, where the tetrahedra share corners to form a network structure of Si–O–Si rings.^57–60^ In amorphous-phase silica glass, the intertetrahedral bridging angles of the ring are widely distributed. This indicates that the network structure of silica glass contains partially energetically unstable structures. In contrast, single-crystal α-quartz consists of 6 and 8 tetrahedral rings, which are energetically stable structures.^61,62^ The etching of SiO_2_ by HF molecules proceeds via a chain reaction that begins with the bond formation between Si and F atoms.^61^ This is caused by the weakening of the chemical bonds around the Si atoms due to electron transfer to F atoms. The fluorination of the energetically unstable parts of silica glass is considered to proceed with a lower reaction barrier energy than that of the stable structural parts. Thus, the number of Si–F bonds that can be formed instantaneously on silica glass exceeded that on α-quartz, as shown in Figure 2c. We ascribed the difference in the maximum etching rates of silica glass and α-quartz to the difference in the densities of the starting points of the fluorination chain reaction in the catalyst-covered areas. By contrast, the internal network structure of α-quartz is altered by heating treatment even at temperatures below the α–β quartz phase transition conditions.^62,63^ This phenomenon is known to degrade the performance of quartz resonators. The crystal structure of α-quartz at the processing temperature ranges was investigated using X-ray diffraction (XRD), as shown in Figure 2d. As the temperature increased, the peak position of α-quartz 110 shifted to a lower angle. This indicates that the intertetrahedral bridging angles of the rings in the quartz change owing to the increase in the lattice spacing caused by thermal expansion. Additionally, the full-width at half-maximum (FWHM) of the XRD peaks broadened as the temperature increased. This suggests that the disordered structures were locally generated in the quartz. The chemical durability of α-quartz is considered to decrease because of the increase of the lattice distance and generation of disordered structures. Therefore, the maximum etching rate of the quartz was obtained under higher temperature conditions than that of the silica glass. Accordingly, the optimal processing temperature for the HF gas-phase catalyst etching of α-quartz is approximately 300 °C, which promotes the formation of Si–F bonds with the active sites of the remaining catalysts. Interestingly, the etching rates of α-quartz and silica glass were almost the same at processing temperatures of >350 °C, as shown in Figure 2a. This is likely because the number of Si–F bonds under the catalyst no longer differed from that of silica glass owing to the thermal decomposition of the catalyst and increase in the energetically unstable regions on the α-quartz. Moreover, lateral etched structures on the microfabricated quartz were not observed after the catalyst etching at 350 °C, as shown in Figure 1c–e. This suggests that Si–F bond formation in the thermally induced unstable structure does not proceed without the presence of a catalyst. This high etching selectivity enables the fabrication of vertical microstructures on α-quartz without requiring plasma sources and hard masks. The etching rate at the optimal processing temperature can be controlled by the HF concentration, as shown in Figure 2e. A high etching rate of 1.5 μm/min was achieved at an HF concentration of 60 vol%. These results indicate that the catalyst etching proceeds via the diffusion of HF molecules. However, the etching rate did not increase linearly with the HF concentration. This nonlinearity is attributed to the hydrolysis of the photoresist caused by H_2_O molecules generated from the etching reaction. Therefore, the increase in hydroxyl groups within the photoresist served as additional catalytic active sites, leading to a further increase in the etching rate. The etching rate also varies depending on the thickness of the photoresist, as shown in Figure 2f. As the photoresist thickness decreased, the etching rate was decreased at both 300 and 350 °C conditions. The difference in etching rates at the same processing temperature is due to differences in the density of catalytic active sites at the photoresist/SiO_2_ interface. Hydroxyl groups in the photoresist are decomposed by ultraviolet (UV)-ozone cleaning prior to the etching process, as shown in Figure 2g. In thinner photoresists, the UV decomposition reaches near the SiO_2_ interfaces. Therefore, the etching rate was influenced by the photoresist thickness.

**Figure 2:**
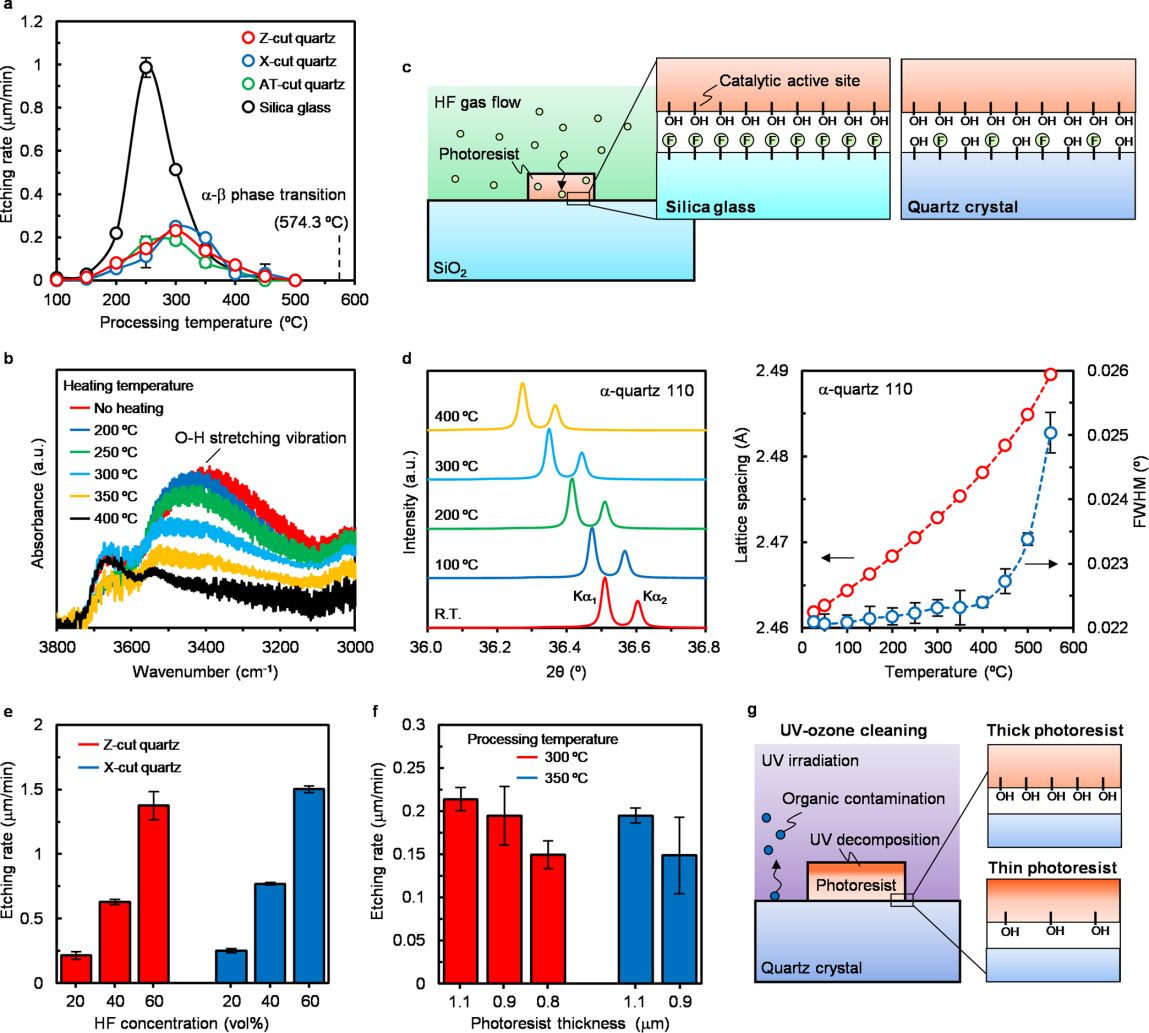
Characterization of HF gas-phase catalyst etching for α-quartz and silica glass. (a) HF gas-phase catalyst etching rates of α-quartz and silica glass at different processing temperatures. The error bars indicate the 3σ standard deviations (*n* = 5). (b) O–H stretching vibrations in the Fourier transform infrared (FT-IR) spectra of catalysts at different heating temperatures. (c) Schematic of the different densities of the starting point of fluorination chain reactions in catalyst-covered areas on silica glass and α-quartz. (d) X-ray diffraction (XRD) pattern around the (110) peak of X-cut quartz at different temperatures (left) and lattice spacing of the X-cut quartz (110) and full-width at half-maximum (FWHM) values of the Kα_1_ peaks at different temperatures (right). The error bars indicate the 3σ standard deviations (*n* = 5). (e) HF gas-phase catalyst etching rates of α-quartz under 300 °C at different HF concentrations. The error bars indicate the 3σ standard deviations (*n* = 5). (f) HF gas-phase catalyst etching rates of X-cut quartz under HF/N_2_ = 20 vol % conditions at different photoresist thickness. The error bars indicate the 3σ standard deviations (*n* = 5). (g) Schematic of the UV decomposition of the photoresist during the UV-ozone cleaning process.

### Characteristics of Microstructures on α-Quartz Fabricated via HF Gas-Phase Catalyst Etching

The vertical microfabrication of α-quartz is typically performed via inductively coupled plasma reactive-ion etching (ICP-RIE). In this study, we compared the ICP-RIE and HF gas-phase catalyst etching for α-quartz using the same patterned photoresist, as shown in Figure 3a. The etching depth for each processing time is plotted in Figure 3b. In the ICP-RIE process, the etching depth did not increase beyond a depth of ∼2.5 μm even when the processing time was extended to >15 min, as shown in Figure 3c. This depth limitation was due to the disappearance of the 1.0-μm-thick photoresist used as a hard mask. In contrast, the depth via the HF gas-phase catalyst etching increased with the processing time, as shown in Figure 3d. A high-aspect vertical microstructure with a depth of over 10 μm was obtained on an α-quartz when the HF gas-phase treatment was performed for 40 min, as shown in Figure 3e. Furthermore, the thickness of the remaining photoresist at the bottom of the trench was constant in the range of 800 to 850 nm after each processing time, as shown in Figure 3f. This result suggests that thin photoresist patterns can be used as catalysts for the vertical microfabrication of α-quartz without depth limitations. However, the thickness of the photoresist before catalyst etching was reduced to less than 1.0 μm due to UV-ozone cleaning prior to the processing.

**Figure 3:**
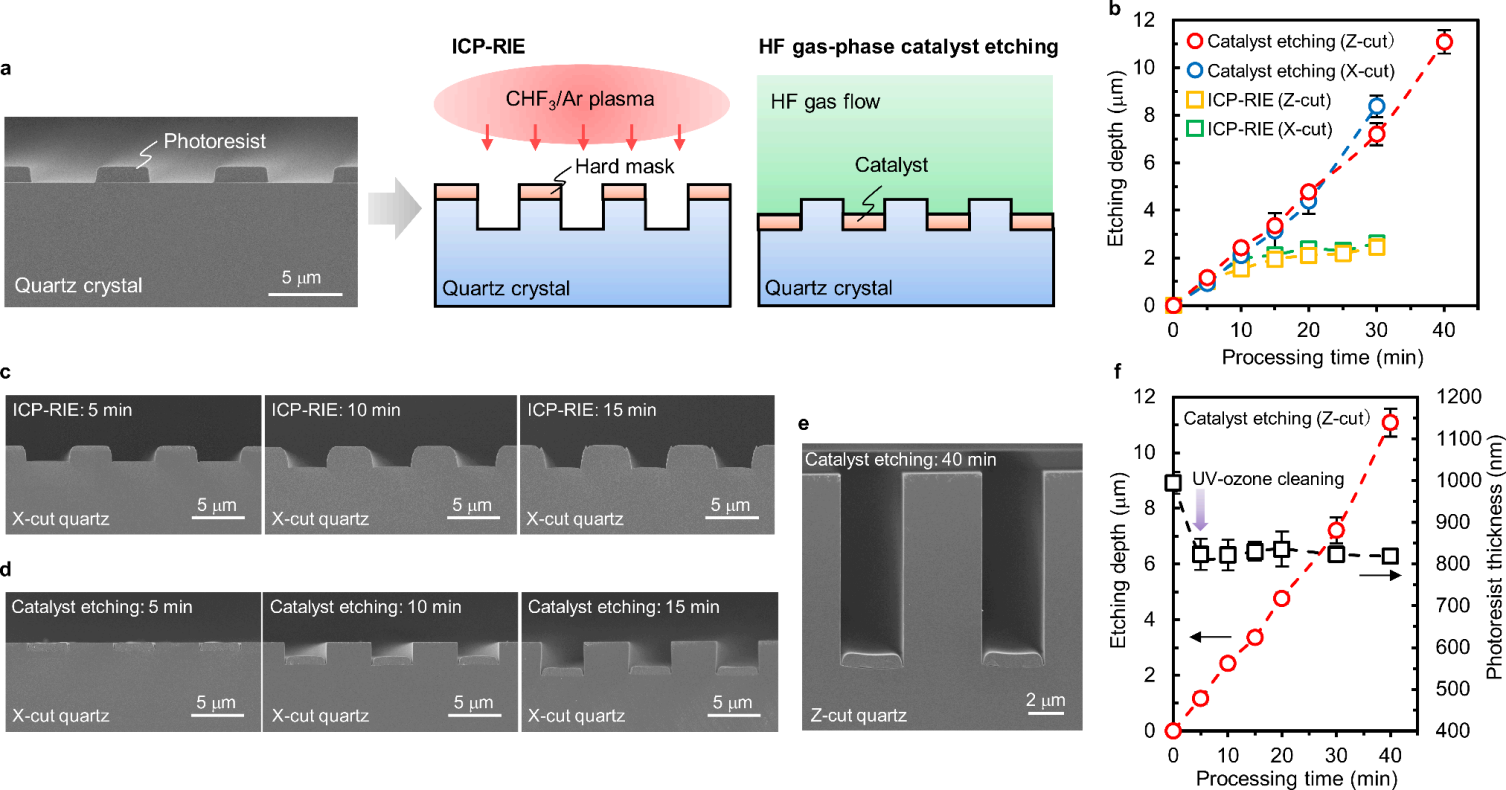
Photoresist patterned area of α-quartz after microfabrication via ICP-RIE and HF gas-phase catalyst etching. (a) Cross-sectional SEM image of a photoresist pattern on α-quartz (left) and schematic of microfabrication via ICP-RIE and HF gas-phase catalyst etching (right). (b) Etching depth of α-quartz upon HF gas-phase catalyst etching at 300 °C and ICP-RIE at different processing times. The error bars indicate the 3σ standard deviations (*n* = 5). (c) Cross-sectional SEM images of X-cut quartz microfabricated via ICP-RIE at different processing times. (d) Cross-sectional SEM images of X-cut quartz microfabricated via HF gas-phase catalyst etching at different processing times. (e) Cross-sectional SEM image of Z-cut quartz microfabricated via HF gas-phase catalyst etching for 40 min. (f) Etching depth and photoresist thickness of Z-cut quartz upon HF gas-phase catalyst etching at 300 °C conditions at different processing times. The error bars indicate the 3σ standard deviations (*n* = 5).

The crystallinity of the etched surface was evaluated through cross-sectional transmission electron microscopy (TEM) and the fast Fourier transform (FFT) of the high-resolution TEM image. The TEM image of the etched surface fabricated via the ICP-RIE indicated that a modified layer was formed to a depth of ∼10 nm from the etched surface, as shown in Figure 4a. The FFT image of the modified layer did not exhibit periodic spot patterns indicative of a crystalline structure, as shown in Figure 4b. This amorphization is attributed to physical ion irradiation of the etched surface during the ICP-RIE. In contrast, the FFT image of the etched surface fabricated via the catalyst etching exhibited periodic spot patterns, indicating the crystallinity of α-quartz, as shown in Figure 4c,d. The chemical state of the etched bottom surface was investigated through X-ray photoelectron spectroscopy (XPS). The Si 2p XPS spectrum of the quartz treated with the ICP-RIE was shifted to a higher binding energy compared with that of unprocessed quartz, as shown in Figure 4e. This suggests that Si–F bonds were formed in the modified layer owing to the ion irradiation during the process.^64^ Moreover, the peak position of the quartz treated with the catalyst etching was almost the same as that of the unprocessed quartz. Therefore, internal damage such as amorphization or chemical-state changes on the etched surface did not occur upon HF gas-phase catalyst etching because the chemical reaction proceeded within the photoresist contact area on the α-quartz.

**Figure 4:**
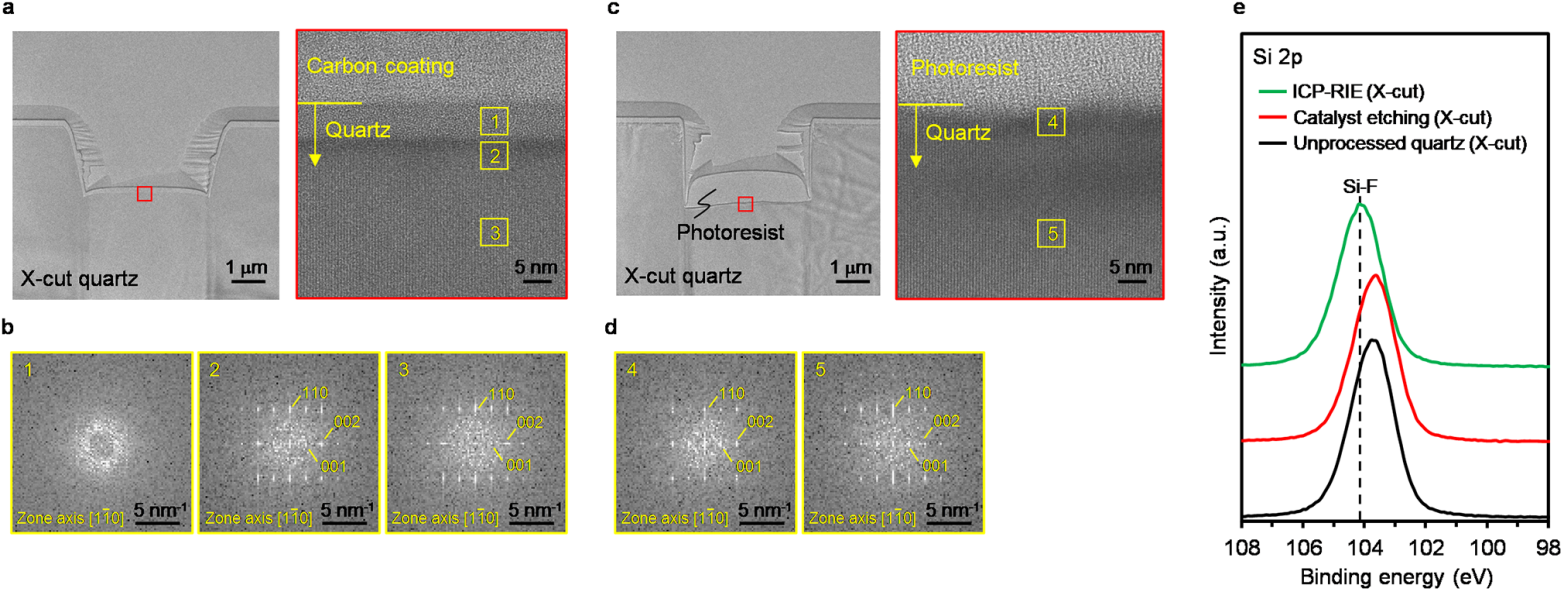
Characterization of etched surfaces subjected to ICP-RIE and HF gas-phase catalyst etching. (a) Cross-sectional transmission electron microscopy (TEM) image (left) and magnified TEM image (right) showing the etched surface on X-cut quartz fabricated via ICP-RIE. (b) Corresponding fast Fourier transform (FFT) pattern for the etched X-cut quartz fabricated via ICP-RIE. (c) Cross-sectional TEM image (left) and magnified TEM image (right) showing the etched surface on X-cut quartz fabricated via HF gas-phase catalyst etching. (d) Corresponding FFT pattern for the etched X-cut quartz fabricated via HF gas-phase catalyst etching. (e) Si 2p XPS spectra of the etched bottom surface of X-cut quartz treated with HF gas-phase catalyst etching and ICP-RIE, along with unprocessed X-cut quartz surface.

Table 1 shows the comparison of α-quartz etching methods: wet etching, ICP-RIE, and HF gas-phase catalyst etching. Wet etching enables batch processing and achieves damage-free microfabrication with infinite selectivity when using a Au/Cr mask. However, the etching profile is highly dependent on the crystal orientation, which limits the shape of microfabrication of α-quartz. In contrast, ICP-RIE achieves microfabrication of α-quartz independent of the crystal orientation. However, the shape of microstructures are tapered due to the selectivity of the mask materials. Additionally, the etched surface is damaged such as the amorphization and chemical-state changes. The HF gas-phase catalyst etching introduced in this study is a novel method that combines the advantages of both wet etching and ICP-RIE. This approach enables vertical etching at higher rates than the conventional processes, regardless of the crystal orientation. Notably, infinite selectivity of the photoresist used as a catalyst has been demonstrated, enabling vertical microfabrication of α-quartz without depth limitations. Furthermore, the etched surface is not damaged because this etching is caused via a chemical reaction with HF molecules. The catalyst etching is a simple process requiring only substrate heating and gas flow control, allowing for the catalyst etching of multiple large wafers in a single process. This process is similar to thermal chemical vapor deposition (CVD), and the uniformity on large wafers is expected to be within 1%, which is equivalent to the performance of thermal CVD.^65–67^

**Table 1:** Comparison of Etching Methods for α-Quartz

Method	Wet etching^31–33^	ICP-RIE^24,37,38^	HF gas-phase catalyst etching
Phase	Liquid	Vacuum plasma	Gas
Critical dimension	<100 nm	<100 nm	<100 nm
Etching rate	0.57–1.26 μm/min (0001)Z	0.5–0.68 μm/min	0.2–1.5 μm/min
	0.002–0.003 μm/min ( 1̅ 100)m		
Etching profile	Crystal anisotropy	Tapered	Vertical
Selectivity	Infinite (Au/Cr)	1.28–2.5 (Photoresist)	Infinite (Photoresist) used as a catalyst
		21 (Cr)	
		30 (Ni)	
Surface damage	Damage-free	Amorphization	Damage-free
		Chemical-state changes	
Wafer processing	Batch process	Single-wafer process	Batch process

## Conclusion

HF gas-phase catalyst etching may help overcome the limitations of the RIE process, which is conventionally used for microfabrication of α-quartz. The novelty of the proposed method is that it allows the vertical microfabrication of α-quartz via HF gas-phase chemical reactions. This approach overcomes the critical problems associated with RIE, such as the depth limit of vertical microfabrication due to the selectivity of the hard masks and the formation of damage layers on the etched surface. Furthermore, we demonstrated vertical microfabrication on Z-, X-, and AT-cut quartz via the catalyst etching, confirming that vertical microfabrication independent of the crystallographic orientations can be realized on α-quartz. The fabrication of damage-free deep vertical microstructures on α-quartz, which is impossible when using conventional etching methods, represents a revolutionary advancement. The crystallinity of α-quartz greatly affects the performance of quartz devices. Therefore, HF gas-phase catalyst etching eliminates various constraints of conventional processes and enables the formation of microstructures on α-quartz with a high degree of design flexibility. The design freedom of microfabrication will play an important role in the development of next-generation quartz-based devices and materials. Additionally, HF gas-phase catalyst etching is a simple process that does not require vacuum chambers, plasma sources, or complex parameter settings. Thus, the proposed technique can be easily applied in various fields by individuals who are not specialized in microfabrication processes.

## Experimental Section

### Sample Preparation and Microfabrication

Optical-grade α-quartz crystal substrates (square; length: 10–20 mm; thickness: 0.5 mm) and synthetic silica glass substrates (square; length: 20 mm; thickness: 0.5 mm), subjected to ultraviolet (UV)–ozone cleaning, were used as the etched substrates. The substrates were treated with hexamethyldisilazane, and a 1.0-μm-thick positive-tone novolac-type photoresist (THMR-iP 5700, Tokyo Ohka Kogyo, Japan) was applied via spin-coating. The photoresist was patterned with periodic 5-μm-wide linear shapes using a maskless photolithography system (DL-1000A2, Nano System Solutions, Japan) and an alkaline developer solution (NMD-3, Tokyo Ohka Kogyo, Japan).

The microfabrication of α-quartz and silica glass was performed via HF gas-phase catalyst etching under atmospheric-pressure gas-phase conditions with a rapid thermal annealing system (VHC-P610, ULVAC, Japan). The gas-flow condition was set at HF/N_2_ = 20 vol% flowing at 5.0 standard liters per minute (SLM). The sample was heated to etching conditions for 2 min, held for 1 min, and then treated with HF/N_2_ gas. UV–ozone cleaning was conducted to remove atomic-level organic contamination from the part of the substrates not covered by the photoresist pattern before HF gas-phase treatment. Anisotropic microfabrication was performed via a conventional dry etching process with an inductively coupled plasma reactive-ion etching (ICP-RIE) system (RIE-101iPH, SAMCO, Japan) with CHF_3_ and Ar gas. The operating ICP/Bias power was set to 300/50 W, and the chamber pressure was 1.0 Pa. The photoresist pattern used for HF gas-phase catalyst etching was used as the hard mask during the ICP-RIE process. After microfabrication, UV–ozone cleaning was performed to remove the photoresist from the sample.

### Characterization

The microstructures of the etched samples were examined by field-emission scanning electron microscopy (SEM; SU8600, Hitachi, Japan). Elemental maps of the samples were obtained via energy-dispersive X-ray spectroscopy (EDS; EMAX-Evo, Horiba, Japan) with an SEM system (SU8000, Hitachi, Japan). The SEM and EDS samples were prepared via sputter-coating with Pt. The surface morphology of the etched surface was evaluated by using atomic force microscopy (AFM; Cypher ES, Asylum Research, USA). The arithmetic mean roughness (Ra) value was obtained from the AFM images. The hydroxyl groups in the catalyst were analyzed via Fourier transform infrared spectroscopy (FT-IR; NICOLET iS50 FT-IR, Thermo Fisher Scientific, USA). The thermally decomposed samples were prepared by using the rapid thermal annealing system. The samples were heated to each treatment temperature for 2 min and then held for 1 min. The gas-flow conditions were N_2_ = 100 vol% flowing at 5.0 SLM. The internal crystal structure of α-quartz was evaluated via X-ray diffraction (XRD; SmartLab, Rigaku, Japan) with Cu Kα radiation. The sample temperature during the XRD analysis was controlled by using a high-temperature attachment (HT 1500, Rigaku, Japan). The crystallographic structures of the etched surface were evaluated through transmission electron microscopy (TEM; JEM-ARM200F, JEOL, Japan) at an accelerating voltage of 200 kV. The TEM samples were prepared through focused ion beam SEM (FIB-SEM; Helios1200, Thermo Fisher Scientific, USA) by using a Ga ion gun. The crystallographic structures on the etched surfaces were determined by using the fast Fourier transform of high-resolution TEM images. To mitigate electron-irradiation-induced amorphization of α-quartz, TEM was conducted with careful optimization of the acquisition conditions. The chemical states of the etched surfaces were evaluated from the Si 2p spectra obtained through X-ray photoelectron spectroscopy (XPS; Quantera II, ULVAC PHI, Japan). The XPS spectra were collected at an incidence angle of 45° over a 100-μm-diameter area on the etched bottom surface.
